# Long-Term Retention of an Intraorbital Metallic Foreign Body Adjacent to the Optic Nerve

**DOI:** 10.1155/2016/3918592

**Published:** 2016-10-12

**Authors:** Andrew N. Siedlecki, Edmund Tsui, Jie Deng, Donald M. Miller

**Affiliations:** ^1^Geisel School of Medicine at Dartmouth, Hanover, NH, USA; ^2^Department of Surgery, Dartmouth-Hitchcock Medical Center, Lebanon, NH, USA; ^3^Department of Ophthalmology, New York University School of Medicine, New York, NY, USA; ^4^Section of Ophthalmology, Dartmouth-Hitchcock Medical Center, Lebanon, NH, USA

## Abstract

We report the case of an asymptomatic 47 year-old male patient who suffered a penetrating wound from a metallic foreign body that became embedded adjacent to the optic nerve for over thirty years, as well as the associated examination, imaging, and fundus photography. Intraorbital metallic foreign bodies can be well tolerated and may not require surgical intervention despite proximity to important structures.

## 1. Introduction

Intraorbital foreign bodies are not uncommon complications following projectile injuries and industrial accidents [1, 2]. Their management is a complex issue requiring input from both the ophthalmologist and patient. While damage caused during the object's travel to the orbit may necessitate surgical intervention, conservative management of inorganic intraorbital foreign bodies should be thoroughly considered. Here, the authors describe the case of a well-tolerated long-term retained metallic foreign body adjacent to the optic nerve without surgical intervention.

## 2. Case Report

A 47-year-old otherwise healthy Caucasian male was evaluated for traumatic subdural hematoma and facial fractures following an altercation. The patient was visually asymptomatic, denying any change in visual acuity, loss of peripheral vision, or diplopia. Past ocular history was notable for a BB gun injury to the left eye at the age of twelve, for which he had never sought medical evaluation. Without any ocular conditions, he had never had an ophthalmological examination. Another medical history included occasional alcohol and tobacco use.

**Figure 1:**
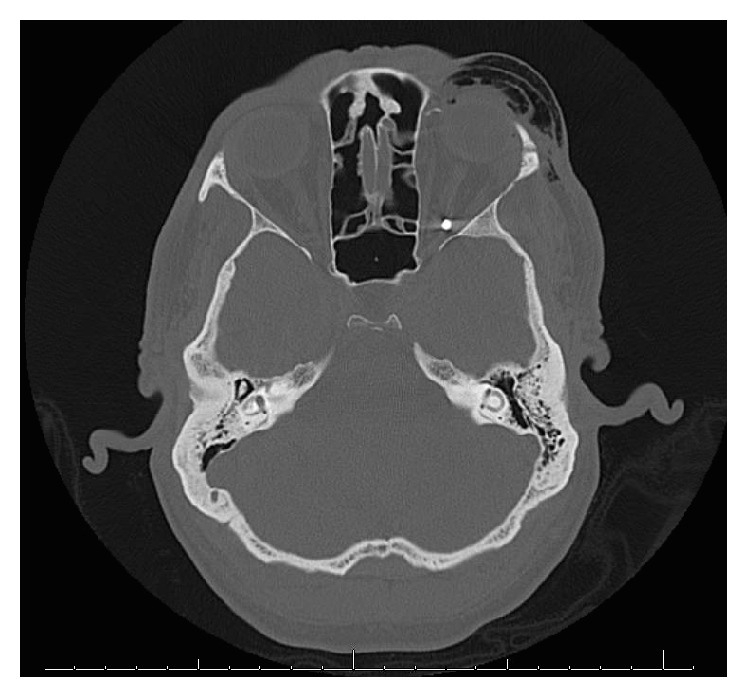
A representative axial head computed tomography image demonstrating an intraorbital metallic foreign body adjacent to the left optic nerve.

A noncontrast head CT showed left orbital floor and lateral wall fractures with associated soft tissue swelling. No evidence of globe injury or muscle entrapment was present; however, a small round metallic foreign body was noted in the left orbital apex abutting the superior aspect of the optic nerve (Figure 1). There were also small posterolateral intraocular calcifications in the left eye.

**Figure 2:**
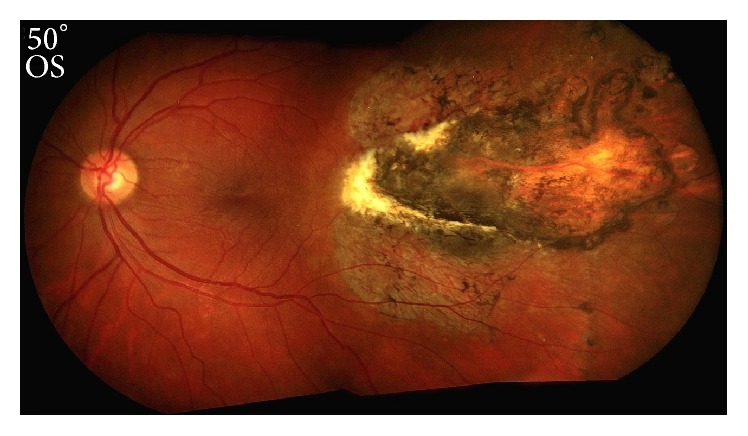
Color fundus photograph of the patient's left eye demonstrating a comet-shaped temporal chorioretinal scar consistent with sclopetaria.

The patient's visual acuity with a reading card was 20/25 in the right and left eyes. Visual fields were full to confrontation in the right eye, but a significant nasal field deficit was present in the left eye. Pupils were equal and reactive without an afferent pupillary defect (APD). Intraocular pressure was 14 mmHg in the right eye and 19 mmHg in the left eye. Elevation of the left eye was mildly restricted, but all other positions of gaze were full bilaterally. Left adnexal edema and ecchymosis as well as temporal subconjunctival hemorrhage of the left eye were present. The remainder of his anterior examination was normal. Fundus examination showed healthy discs, vessels, and maculae bilaterally; however, a large elongated peripheral temporal chorioretinal scar consistent with sclopetaria was noted in the left eye (Figure 2).

Given his lack of symptomatology and uneventful ocular history, a conservative therapeutic approach with periodic observation was recommended. He was referred for outpatient surgical management of his facial fractures following resolution of adnexal edema.

## 3. Discussion

Our patient presented with well-tolerated long-term retention of a metallic foreign body adjacent to the optic nerve without surgical intervention. The patient's chorioretinal scar and visual field deficit are consistent with sclopetaria following projectile injury at age twelve. His visual acuity has been stable without evidence of optic neuropathy. Given the lack of bothersome symptomatology, conservative management was recommended.

Removal of intraorbital foreign bodies is a complex issue requiring assessment of the composition, location, and size of the penetrating body, as well as associated signs and symptoms [1–9]. Organic foreign bodies should be expeditiously removed due to significant risk of orbital inflammation and infection [2, 3, 8, 9]. Generally, inorganic intraorbital foreign bodies are better tolerated. Exceptions include copper materials, which have been reported to cause purulent inflammation, iron, which can cause siderosis, and lead, which can cause systemic toxicity [3, 7, 9, 10]. Symptomatic inorganic foreign bodies generally merit surgical exploration; however, in the absence of obvious signs or symptoms, location and size of metallic foreign bodies are central in the decision to surgically intervene, though perioperative risks can offset putative benefits [1–3, 8, 9].

When evaluating intraorbital foreign bodies, consideration must be given to the mechanism by which the object was introduced. While entry through the lids or fornices can occur, intraorbital foreign bodies more often involve a perforating globe injury. A retrospective analysis of 182 ocular missile injuries identified several risk factors for poor prognosis: poor initial visual acuity (worse than 20/800), presence of APD, wounds involving the sclera, injuries extending posterior to the rectus muscle insertions, wounds greater than 10 mm in length, lens subluxation or expulsion, severe vitreous hemorrhage obscuring the disc and retinal vessels, and retained intraocular fragments [5].

A retrospective study reported the outcomes of 50 eyes with conservatively managed metallic intraorbital foreign bodies [10]. Thirty-seven were located posterior to the globe, three of which were at the orbital apex. For these apical foreign bodies, the visual acuities at presentation were no light perception (NLP) and remained so following steroid treatment. While 93% of all patients in this study had improved vision following steroids, patients presenting with acuity at 20/200 or worse were highly likely to remain as such and few cases showed improvement to 20/50 or better. Complications occurred in only 5% of cases. Generally, patients requiring surgical intervention had worse visual outcomes.

A retrospective analysis of five cases with intraorbital foreign bodies adjacent to the optic nerve suggests that their retrieval can significantly reduce psychological morbidity [11]. These patients presented within a month from their injury with significant loss of vision ranging from hand motion (HM) to NLP and also reported symptoms of anxiety and/or insomnia. After surgery, three patients remained NLP or HM and two had visual acuities of 20/500 and 20/1000; however, all patients reported improved psychological symptoms.

Protocols for intraorbital foreign body management recommend that posteriorly located foreign bodies without overt complications should be managed nonsurgically, whereas anteriorly located foreign bodies are safer to remove [1–3]. While visual outcomes are unlikely to improve, removal allows for future magnetic resonance imaging. Ultrasonography may also be a useful adjunct to diagnose or monitor intraorbital foreign bodies [12]. Surgery for asymptomatic inorganic foreign bodies in the posterior orbit has significantly increased risk of perioperative morbidity without consistently demonstrated clinical benefits [1, 2]. While traumatic optic neuropathy is often seen in these cases, patients presenting with poor visual acuity in this setting are unlikely to recover significant visual function with surgical extraction [1, 5].

In conclusion, given the asymptomatic long-term retention of this patient's metallic foreign body, surgical intervention was not indicated despite the object's proximity to the optic nerve. The literature suggests that consideration of size, composition, location, and presenting symptoms are integral to the decision regarding surgical retrieval.
